# Financial Exploitation Vulnerability and Social Connectedness in Middle-Aged and Older Adults Without Dementia

**DOI:** 10.1093/geroni/igaf122.107

**Published:** 2025-12-31

**Authors:** Daisy Noriega-Makarskyy

**Affiliations:** University of Southern California, Los Angeles, California, United States

## Abstract

Financial exploitation of older adults is an understudied but widespread phenomenon in the United States. Previous research examining the association between social functioning and financial exploitation vulnerability suggests that social embeddedness may be protective against financial exploitation. It is not clear, however, whether relationship depth and/or social network diversity (i.e., having many different social roles) drives this protective effect. This study aimed to examine the relationship between aspects of social connectedness (i.e., social network diversity and relationship depth) and financial exploitation vulnerability amongst community-dwelling adults aged 50 or older. One hundred eighteen individuals completed a laboratory visit consisting of questionnaires assessing relationship depth (i.e., Interpersonal Support Evaluation List), social network diversity (i.e., Social Network Index Total Social Roles), and financial exploitation vulnerability (i.e., Perceived Financial Vulnerability Scale; PFVS). Hierarchical linear regressions separately tested associations between financial exploitation vulnerability, relationship depth, and social network diversity. After covarying for demographics, cognitive, and psychological variables, results showed a significant negative association between financial exploitation vulnerability and relationship depth (B(SE) = -.09(.03), p = .004). Subscale analysis indicated that a strong sense of belonging was negatively associated with financial exploitation vulnerability (B(SE) = -.28(.06), p < .001). In contrast, financial exploitation vulnerability was not significantly associated with the number of distinct social roles in one’s network (B(SE) = -.02(.11), p = .85), or with other relationship depth subscales. These findings suggest potential benefits of fostering close interpersonal relationships in middle and older adulthood, such as reduced vulnerability to financial exploitation.

